# Indirect Treatment Comparison of Riociguat Replacement Therapy and Selexipag Add-on Therapy in Patients With Pulmonary Arterial Hypertension: Results From a Systematic Review

**DOI:** 10.31083/RCM46524

**Published:** 2026-05-27

**Authors:** Ji-Eun An, Jahyun Cho, Min Ju Kim, Ah-Yeon Lee, Woo-Jeong Sim, Gyeong-U Hong, Soo Hyun Lee, Dong-Sook Kim, Sun-Young Yi, Kyung-Min Lee, Su-Yeon Yu

**Affiliations:** ^1^College of Pharmacy, Kangwon National University, 24341 Kangwon, Republic of Korea; ^2^Graduate School of Public Health, Seoul National University, 08826 Seoul, Republic of Korea; ^3^Department of Medical Information, School of Nursing and Health, Kongju National University, 32588 Kongju, Republic of Korea; ^4^Department of Health Administration, School of Nursing and Health, Kongju National University, 32588 Kongju, Republic of Korea; ^5^Department of Market Access, Bayer Korea, 07335 Seoul, Republic of Korea

**Keywords:** pulmonary hypertension, riociguat, selexipag, systematic review, comparative effectiveness research

## Abstract

**Background:**

Despite standard combination therapy with endothelin receptor antagonists (ERAs) and phosphodiesterase-5 inhibitors (PDE5is), many patients with pulmonary arterial hypertension (PAH) show inadequate therapeutic responses. Riociguat (a soluble guanylate cyclase stimulator) and selexipag (a prostacyclin receptor agonist) are both approved as next-step therapies; however, their comparative effectiveness and safety remain unknown due to the lack of head-to-head trials. We aimed to compare the therapeutic effects of riociguat replacement and selexipag add-on therapy through an indirect treatment comparison.

**Methods:**

Randomized controlled trials (RCTs) involving patients with PAH receiving either riociguat or selexipag were identified through a systematic search of PubMed, EMBASE, and the Cochrane Library up to 04 November 2025. This systematic review adhered to the Preferred Reporting Items for Systematic Reviews and Meta-Analyses guideline. Study quality was assessed with Cochrane's Risk of Bias 2.0 tool. Indirect treatment comparisons using Bucher's method were conducted within a common comparator (ERA + PDE5i) framework.

**Results:**

Three RCTs (four publications) were included: REPLACE, GRIPHON (main and post-hoc analyses), and a phase II trial. The overall risk of bias was low, except for the phase II trial, which had unclear risk due to its small sample size. Indirect comparisons showed no significant differences between the therapies for any outcome. The hazard ratio for clinical worsening was 0.167 (95% confidence interval [CI]: 0.0019–1.495, *p* = 0.1066). Mean differences for 6-min walk distance and N-terminal pro–B-type natriuretic peptide were 10.64 m (95% CI: –9.158 to 30.438, *p* = 0.2920) and –46.62 pg/mL (95% CI: –307.826 to 214.586, *p* = 0.7263). The relative risk of overall adverse events was 1.07 (95% CI: 0.90–1.27, *p* = 0.453). Subgroup analyses of patients receiving baseline ERA + PDE5i therapy and those classified as World Health Organization functional class III also showed no significant differences.

**Conclusions:**

We found no significant differences between riociguat replacement and selexipag add-on therapy. These findings provide comparative data to help clinicians and patients make informed treatment decisions. Further head-to-head trials are needed to confirm comparative effectiveness. This review adhered to PRISMA 2020 guidelines.

**The PROSPERO Registration:**

CRD42024524391 https://www.crd.york.ac.uk/PROSPERO/view/CRD42024524391.

## 1. Introduction

Pulmonary hypertension (PH) is defined as a mean pulmonary arterial pressure (mPAP) ≥20 mmHg at rest, measured by right heart catheterization. Clinically, PH is classified into five groups based on pathophysiology and treatment strategy [1].

Group 1, or pulmonary arterial hypertension (PAH), is defined as pre-capillary PH that meets specific hemodynamic criteria and occurs in the absence of significant lung or thromboembolic disease. PAH include idiopathic PAH, hereditary PAH, drug- and toxin-induced PAH, disease-associated PAH, and PAH in patients with a long-term response to calcium channel blockers. PAH leads to right ventricular dysfunction, resulting in symptoms such as dyspnea and decreased exercise capacity. It is a life-threatening condition with increased mortality if not treated effectively [2].

The 2022 European Society of Cardiology/European Respiratory Society (ESC/ERS) guidelines lowered the diagnostic threshold for pulmonary hypertension from mPAP ≥25 mmHg, as defined in the 2015 ESC/ERS guidelines, to ≥20 mmHg and refined risk stratification from a three-tier to a four-tier model, allowing more granular assessment of parameters such as 6-minute walk distance (6MWD), World Health Organization functional class (WHO FC), and N-terminal pro–B-type natriuretic peptide (NT-proBNP) to guide treatment intensity [1, 3].

In South Korea, the 2020 Korean Society of Cardiology/Korean Academy of Tuberculosis and Respiratory Diseases (KSC/KATRD) pulmonary hypertension guideline is being updated to align with the 2022 ESC/ERS recommendations, emphasizing earlier detection and individualized escalation of PAH therapy within both international and domestic practice frameworks [4, 5].

PAH pharmacotherapy recommended in the 2022 ESC/ERS guidelines and the 2020 Korean PH guideline targets three main pathways: endothelin receptor antagonists (ERAs), the nitric oxide–cyclic guanosine monophosphate axis (phosphodiesterase-5 inhibitors [PDE5i] and soluble guanylate cyclase [sGC] stimulators), and prostacyclin (PC) signaling. These agents are used alone or in combination to promote vasodilation and inhibit vascular remodeling. The 2022 ESC/ERS guidelines recommend initial oral combination therapy with ERA + PDE5i for most low- to intermediate-risk patients, with consideration of rapid escalation to triple therapy in higher-risk groups [1].

Despite optimized ERA + PDE5i combination therapy, a substantial proportion of patients remain symptomatic or at intermediate–high risk, prompting consideration of additional pathway modulation.

Riociguat, an sGC stimulator, enhances cyclic guanosine monophosphate production and has demonstrated efficacy in patients with WHO FC II–III PAH and chronic thromboembolic pulmonary hypertension (CTEPH) [6], whereas selexipag, an oral IP prostacyclin receptor agonist, selectively augments prostacyclin signaling and is approved for WHO FC II–III PAH as mono- or combination therapy with ERA and/or PDE5i [7]. In clinical practice, these agents are often positioned differently in the treatment algorithm for patients already receiving ERA + PDE5i. Riociguat is typically considered as a “replacement” strategy for PDE5i non-responders, providing mechanistic substitution within the NO–sGC–cGMP pathway, whereas selexipag is used as an “add-on” to expand pathway coverage by activating the prostacyclin axis in partial responders. Thus, clinicians face a real-world decision: in a patient on ERA + PDE5i who remains inadequately controlled, should they switch from PDE5i to riociguat (replacement) or add selexipag on top of ERA + PDE5i (add-on), recognizing that these approaches differ in pathophysiology, pill burden, tolerability, and health system implications [4, 8].

The Korean Health Insurance system covers ERA drugs (ambrisentan, bosentan, and macitentan), PDE5is (sildenafil), and PCs (selexipag, treprostinil, and iloprost) for PAH treatment. Coverage begins with monotherapy and advances to dual therapy if treatment goals are not met, followed by triple therapy when necessary. Of the oral PCs, only selexipag is reimbursed [9]. Thus, introducing riociguat as an alternative pathway-targeted agent for patients treated with PDE5i + ERA is needed, which requires a comparative analysis of the efficacy and safety of riociguat replacement versus selexipag add-on therapy.

Comparing riociguat replacement and selexipag add-on therapies is essential to optimize therapeutic strategies for patients with PAH receiving PDE5i + ERA combination therapy. Randomized controlled trials (RCTs) have demonstrated the efficacy and safety of each drug independently; however, direct comparative studies are lacking. In this study, we aimed to address this gap by conducting an indirect comparison through a systematic review and meta-analysis, providing clinical evidence to guide optimal treatment strategies and support future updates to Korean guidelines.

## 2. Literature Review

We conducted an indirect treatment comparison (ITC) analysis through a systematic literature review and meta-analysis of RCTs to evaluate the efficacy of riociguat replacement versus selexipag add-on therapy in patients with PAH receiving PDE5i + ERA combination therapy. This analysis followed the recommendations of the Cochrane Handbook and Preferred Reporting Items for Systematic Reviews and Meta-Analyses (PRISMA 2020) guidelines (**Supplementary Table 1**). The systematic review protocol was registered in the International Prospective Register of Systematic Reviews (PROSPERO; CRD42024524391).

### 2.1 Systematic Literature Review and Meta-analysis

#### 2.1.1 Literature Search 

A literature search was conducted on 04 November 2025, using the PubMed, EMBASE, and Cochrane Library databases. The search followed a Participants/Intervention/Comparator/Outcome (PICO) strategy to identify relevant RCTs evaluating riociguat replacement or selexipag add-on therapy. The PICO/study design criteria were defined as follows: Participants (P): adult patients with PAH receiving baseline ERA + PDE5i combination therapy; Intervention (I): riociguat replacement therapy (switching PDE5i to riociguat in the existing ERA + PDE5i regimen) or selexipag add-on therapy (adding selexipag to existing ERA + PDE5i therapy); Comparator (C): placebo or comparator within the common comparator framework of ERA + PDE5i ± placebo; Outcome (O): composite endpoint of clinical worsening (improvement or worsening), changes in 6MWD, NT-proBNP, improvement in WHO FC, and safety endpoints (overall and serious adverse event rates); Study Design (S): RCTs only (Table 1).

**Table 1.** **PICO strategy**.

	Details
Patients	Patients with pulmonary arterial hypertension receiving ERA + PDE5i
Interventions	Riociguat replacement therapy (Riociguat ± ERA)
	Selexipag add-on therapy (Selexipag ± ERA ± PDE5i)
Comparators	Placebo or comparator within the common comparator framework of ERA + PDE5i ± placebo
Outcomes	(1) Composite endpoint of clinical worsening
	(2) 6-min walking distance
	(3) N-terminal prohormone of brain natriuretic peptide
	(4) WHO functional level
	(5) Safety: overall adverse events, serious adverse event
Study Design	Randomized controlled trial

The composite endpoint was defined as the time to first morbidity or mortality event, including disease progression, PAH worsening resulting in hospitalization, initiation of parenteral prostanoid therapy or long-term oxygen therapy, need for lung transplantation or balloon atrial septostomy, or death from any cause.

Search terms included “pulmonary arterial hypertension” OR “PAH” AND (“riociguat” OR “selexipag”) AND “randomized controlled trial”, using appropriate Medical Subject Headings or Emtree terms for each database (**Supplementary Table 2**).

#### 2.1.2 Study Selection 

The inclusion criteria were: (1) RCTs involving patients with PAH receiving either riociguat or selexipag; (2) presence of a comparator (placebo or active comparator); and (3) evaluation of clinical efficacy (proportion of patients experiencing clinical events, 6MWD changes, NT-proBNP, or WHO FC improvement) or safety (overall and serious adverse events). Studies that did not meet these criteria were excluded. All members of the study team participated in the initial (abstract and title) and secondary (full-text) review, with two independent reviewers assigned per article, and any disagreements were resolved through discussion among the full team. EndNote 20 (Clarivate Analytics, Philadelphia, PA, USA) and Covidence software (Veritas Health Innovation, Melbourne, VIC, Australia) were used in this study.

#### 2.1.3 Data Extraction 

Data were primarily extracted by one reviewer and verified by a second reviewer, with all study team members participating in this process. Extracted data included basic study information (authors, publication year, trial name, and journal), study design (randomization, blinding, and follow-up duration), patient characteristics (age, sex, PAH etiology/severity, baseline therapies, WHO FC, 6MWD, and NT-proBNP), intervention and comparator details (dosage and administration period), efficacy outcomes (6MWD, clinical event rates, NT-proBNP, and WHO FC) and safety outcomes (overall and serious adverse events). Meta-analyses using a random-effects model were performed for outcomes reported in two or more studies using Review Manager 5.4. (The Cochrane Collaboration, Copenhagen, Denmark) and the Indirect Treatment Comparison software (version 1.0; Canadian Agency for Drugs and Technologies in Health, Ottawa, ON, Canada) was used to produce estimates of the relative clinical utility effect through indirect comparison. In addition, forest plots were generated to visually summarize relative treatment effects and 95% CIs for the primary and secondary outcomes (clinical worsening, 6MWD, NT-proBNP, WHO FC improvement, and adverse events) using Python (version 3.9; Python Software Foundation, Wilmington, DE, USA).

#### 2.1.4 Quality Assessment

Study quality was assessed using the Cochrane Risk of Bias 2.0 tool (RoB 2; The Cochrane Collaboration, London, UK), evaluating random sequence generation, allocation concealment, blinding of participant and investigators, outcome assessor blinding, attrition bias, and selective reporting bias. Two reviewers independently assessed the risk of bias for each study, with discrepancies resolved by consensus with a third reviewer. Similarity in patient populations and study design among the selected trials for indirect comparison was confirmed (**Supplementary Table 3**, **Supplementary Fig. 1**).

### 2.2 Indirect Comparison 

Qualitative comparisons of extracted outcomes from selected studies were summarized in tables and narratives. Indirect comparative analyses were then performed based on the similarity of outcome measures across studies.

Indirect comparison used RCT-reported outcomes comparing ‘ERA + PDE5i vs. ERA + riociguat’ and ‘ERA + PDE5i vs. ERA + PDE5i + selexipag’, with ‘ERA + PDE5i ± placebo’ as a common comparator group. The primary outcome was the composite endpoint of clinical worsening, while the secondary outcomes were changes in 6MWD, NT-proBNP, WHO FC improvement, and overall and serious adverse events. Clinical worsening was compared using hazard ratios (HRs). The GRIPHON study had a longer follow-up of 36 months for clinical worsening compared with the 24-week follow-up in the REPLACE study, indirect comparison was considered feasible. The GRIPHON study estimated HRs using a proportional hazards model, which assumes that the HR remains constant over time. Under this assumption, the HR at 36 months is considered equivalent to the HR at 6 months, allowing a valid comparison despite differences in follow-up duration.

Continuous variables, such as changes in 6MWD and NT-proBNP from baseline at 6 months, were evaluated by mean differences (MDs) and categorical variables (WHO FC improvement and adverse event rates) by risk ratios (RRs). The null hypothesis assumed no difference between riociguat replacement and selexipag add-on therapy, and the alternative hypothesis proposed a significant difference, with a two-sided significance level of 5%.

The primary analysis included all selected RCTs, with sensitivity analyses conducted to assess the robustness of the indirect comparison. First, because the phase II selexipag trial by Simonneau (2012) had a very small sample size [10], markedly higher NT-proBNP levels, and a different assessment time point (week 17), it was included in the primary analysis but excluded in sensitivity analyses, in which a 1:1 indirect comparison was repeated using only REPLACE and GRIPHON. Second, to minimize differences in baseline risk and background therapy, anchored Bucher indirect comparisons were additionally performed as a sensitivity analysis using GRIPHON patients selected to match the baseline characteristics of REPLACE patients, including those receiving ERA + PDE5i at baseline and, within this population, those classified as WHO FC III. Potential issues of homogeneity and exchangeability were assessed by comparing individual patient characteristics across studies. ITCs were conducted using the Canadian Agency for Drugs and Technologies in Health (CADTH) ITC software, which applies anchored indirect comparisons based on the Bucher method [11].

## 3. Results

### 3.1 Systematic Review 

For selexipag add-on therapy in patients with PAH receiving background PDE5i + ERA, 180 articles from PubMed, 165 from the Cochrane Central Library, and 428 from EMBASE were identified. After removing duplicates, 606 articles were screened, and six candidate studies underwent full-text review. The TRACE studies (Howard *et al*., 2023 [12]; Rehman *et al*., 2025 [13]) were excluded from the final indirect comparison because their inclusion criteria allowed patients receiving soluble guanylate cyclase (sGC) stimulators as part of combination therapy. Consequently, clinical outcomes in these trials may have reflected the therapeutic effects of riociguat, introducing confounding. Ultimately, three selexipag studies were retained for indirect comparison: the GRIPHON phase III trial [14, 15] and a phase II trial [10].

**Fig. 1.**
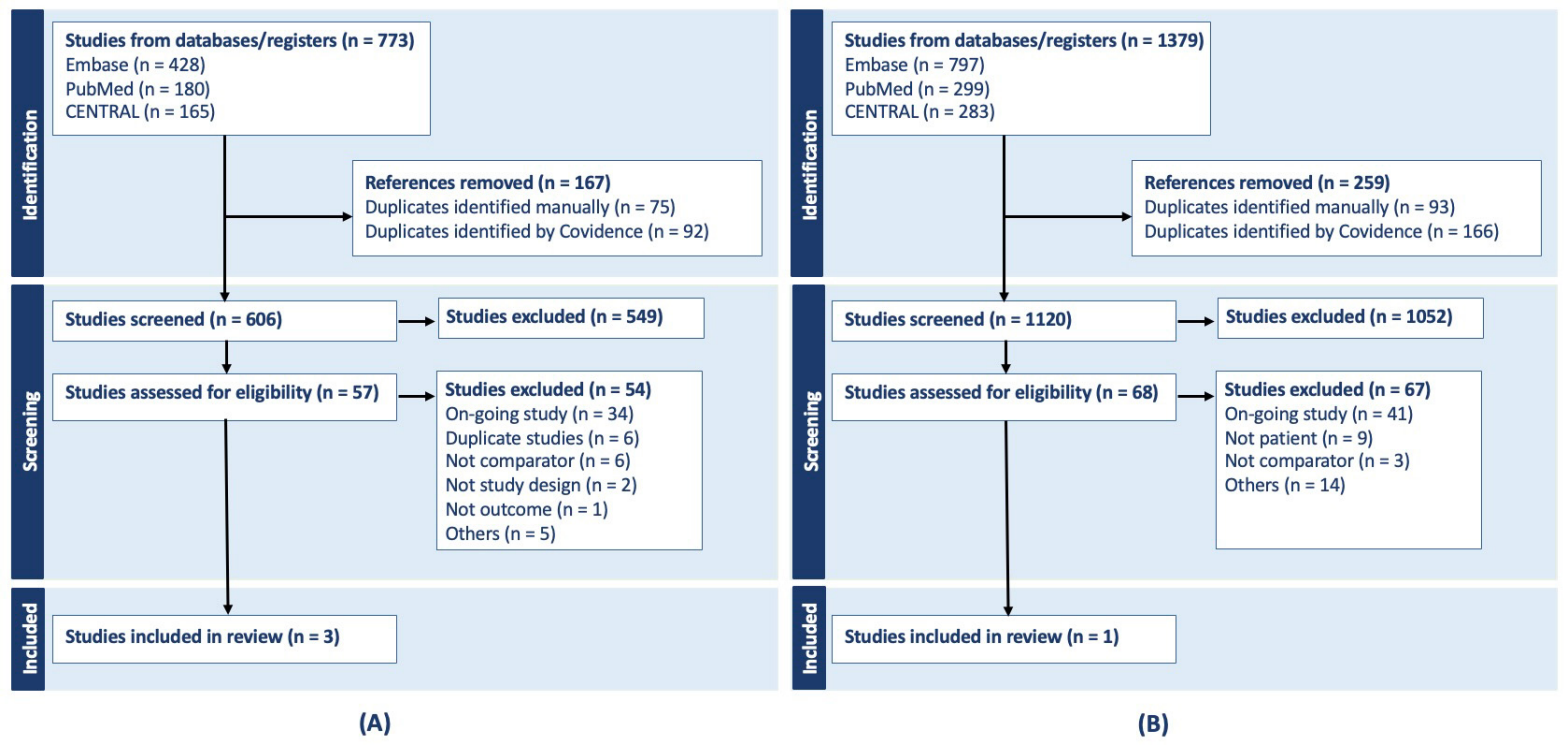
**PRISMA flowcharts for selexipag (A), PRISMA flowchart of riociguat (B)**. PRISMA, Preferred Reporting Items for Systematic Reviews and Meta-Analyses.

For riociguat replacement therapy in patients with PAH on PDE5i + ERA combination, 299 articles from PubMed, 283 from the Cochrane Library, and 797 from EMBASE were identified. After removing duplicates, 1120 articles were screened, and two RCTs were considered potentially relevant. Both Benza *et al*. (2024) [16] riociguat and Benza *et al*. (2024) [17] selexipag stratified patients according to the REVEAL Lite 2 risk score; however, because they evaluated different outcome measures, they could not be incorporated into the Bucher-type indirect comparison. Similarly, the Channick *et al*. (2025) [18] analysis reported age-stratified outcomes in the overall GRIPHON population, but age-specific data were not available from REPLACE, precluding further anchored indirect comparison. Consequently, only the REPLACE phase III trial [1] was included as the riociguat anchor study in the final ITC framework (Fig. 1).

The risk of bias assessment revealed no significant overall bias. All studies used intention-to-treat analyses, addressing missing with either last observation carried forward or worst-value replacement methods. The REPLACE and GRIPHON studies conducted survival analyses with censoring to enhance data integrity. The REPLACE study was open-label; however, potential bias was mitigated by assigning independent reviewers at each study site to ensure adherence to the study protocol. The risk of bias in the Simonneau (2012) Phase II study was uncertain due to its small sample size and unclear protocol verification [10]. This study was included in the primary indirect comparison but excluded from sensitivity analyses (**Supplementary Table 3**).

The mean age of the study populations was similar: REPLACE (49.4 ± 16.2 years) and GRIPHON (48.2 ± 15.19 to 54.8 ± 16.8 years). Both studies included high proportions of females and patients with idiopathic PAH (female: REPLACE 74%, GRIPHON 79.9–81.8%; idiopathic PAH: REPLACE 62%, GRIPHON 54.4–72.7%).

The REPLACE study exclusively enrolled patients in WHO FC III, while GRIPHON included 51–54% of patients in WHO FC III. Despite this difference in severity classification, average 6MWD and NT-proBNP levels were comparable, supporting study compatibility: 6MWD (REPLACE 374 ± 60 m vs. GRIPHON 359.7 ± 80.97 m to 396.2 ± 71.4 m) and NT-proBNP (REPLACE 290 [138–863] pg/mL vs. GRIPHON 514.5 [166.0–1356.0] ng/L to 1601.4 ± 2443.0 pg/mL). However, NT-proBNP levels were 4–8 times higher than in other study populations, likely because the small sample size allowed outliers to skew the mean. Therefore, this study was included in the primary analysis but excluded from sensitivity analyses.

Patients receiving baseline ERA + PDE5i therapy comprised 71–72% of the REPLACE study population and 31.2–33.8% of the GRIPHON population. However, the GRIPHON post-hoc analysis [15] focused on patients exclusively treated with ERA + PDE5i at baseline, making patient characteristics comparable between the studies. Dosages in all included studies were within authorized therapeutic ranges. GRIPHON had a 36-month follow-up; however, outcomes at 26 weeks were used for indirect comparison analyses (Table 2, Ref. [1, 10, 12, 13, 14, 15, 16, 17, 18]).

**Table 2.** **Baseline characteristics of participants in candidate clinical trials for indirect comparisons**.

Trial (Year)		Riociguat switch group	Control group	Selexipag add-on group	Final RCTs excluded in the indirect treatment comparison
Humbert (2023) [1]; Benza (2024) [16] riociguat study (REPLACE)					Humbert (2023) (REPLACE) [1]
	Treatment	Riociguat ± ERA (n = 111)	PDE5i ± ERA (n = 115)		
	Age, Mean ± SD	49.4 ± 16.2 years	49.1 ± 15.7 years		
	Female	74%	83%		
	WHO Functional Class	III 100%	III 100%		
	Asian	15%	17%		
	PAH Etiology	Idiopathic 62%	Idiopathic 65%		
		Heritable 4%	Heritable 4%		
		Drug-/toxin-associated 1%	Drug-/toxin-associated 4%		
		Connective tissue disease-associated 22%	Connective tissue disease-associated 17%		
		Congenital heart disease-associated 5%	Congenital heart disease-associated 6%		
		Portopulmonary 6%	Portopulmonary 5%		
	Background Therapy	ERA 71% (bosentan 17%, ambrisentan 27%, macitentan 27%)	ERA 72% (bosentan 18%, ambrisentan 26%, macitentan 28%)		
	6-MWD, Mean ± SD	374 ± 60 min	367 ± 62 min		
	NT-proBNP, Median (IQR)	290 (138–863) pg/mL	395 (166–1608) pg/mL		
Sitbon (2020) [14]; Benza (2024) [17] slexipag study; Channick (2025) [18] (GRIPHON)					Sitbon (2020) (GRIPHON) [14]
	Treatment		Placebo ± ERA ± PDE5i (n = 574)	Selexipag ± ERA ± PDE5i (n = 582)	
	Age, Mean ± SD		47.9 ± 15.55 years	48.2 ± 15.19 years	
	Female		80.1%	79.6%	
	WHO Functional Class		I 0.9%	I 0.7%	
			II 43.8%	II 47.7%	
			III 54.0%	III 51.0%	
			IV 1.4%	IV 0.5%	
	Asian		19.4%	20%	
	PAH Etiology		Idiopathic 57.9%	Idiopathic 54.4%	
			Heritable 2.2%	Heritable 2.3%	
			Drug-/toxin-associated 1.7%	Drug-/toxin-associated 3.0%	
			Connective tissue disease-associated 28.7%	Connective tissue disease-associated 29.1%	
			Congenital heart disease-associated 8.6%	Congenital heart disease-associated 10.5%	
			HIV-associated 0.9%	HIV-associated 0.9%	
	Background Therapy		ERA 13.1%	ERA 16.4%	
			PDE5i 31.8%	PDE5i 32.9%	
			ERA+PDE5i 33.8%	ERA+PDE5i 31.2%	
	6-MWD, Mean ± SD		348.0 ± 83.23 min	374 ± 60 min	
	NT-proBNP, Median (IQR)		470.0 (170.0–1359.0) pg/mL	514.5 (166.0–1356.0) pg/mL	
Coghlan (2018) [15] (GRIPHON)					Coghlan (2018) [15] (GRIPHON with patients receiving PDE5i + ERA)
	Treatment		Placebo + ERA + PDE5i (n = 179)	Selexipag + ERA + PDE5i (n = 197)	
	Age, Mean ± SD		50.7 ± 14.24 years	50.6 ± 15.00 years	
	Female		79.2%	79.9%	
	WHO Functional Class		II 30.7%	II 30.5%	
			III 68.2%	III 67.5%	
			Others 1.1%	Others 2.0%	
	Asian		8.6%	6.7%	
	PAH Etiology		Idiopathic 59.9%	Idiopathic 59.2%	
			Heritable 4.6%	Heritable 5.0%	
			Drug-/toxin-associated 1.0%	Drug-/toxin-associated 6.7%	
			Connective tissue disease-associated 28.4%	Connective tissue disease-associated 22.3%	
			Congenital heart disease-associated 5.1%	Congenital heart disease-associated 5.6%	
			HIV-associated 1.0%	HIV-associated 1.1%	
	Background Therapy		ERA+PDE5i 100%	ERA+PDE5i 100%	
	6-MWD, Mean ± SD		358.7 ± 79.73 min	359.7 ± 80.97 min	
	NT-proBNP, Median (IQR)		N/A	N/A	
Simonneau (2012) [10] (Selexipag Phase 2)					Simonneau (2012) [10] (Selexipag Phase 2)
	Treatment		Placebo ± ERA ± PDE5i (n = 33)	Selexipag ± ERA ± PDE5i (n = 10)	
	Age, Mean ± SD		53.8 ± 16.3 years	54.8 ± 16.8 years	
	Female		80%	81.8%	
	WHO Functional Class		II 20%	II 45.5%	
			III 80%	III 54.5%	
	Asian		N/A	N/A	
	PAH Etiology		Idiopathic 70%	Idiopathic 72.7%	
			Heritable 10%	Heritable 3%	
			Connective tissue disease 20%	Drug-/toxin 6.1%	
				Connective tissue disease 12.1%	
				Congenital heart disease 6.1%	
	Background Therapy		ERA 40%	ERA 36.4%	
			Sildenafil 30%	Sildenafil 27.2%	
			ERA+Sildenafil 30%	ERA+Sildenafil 36.4%	
	6-MWD, Mean ± SD		350.3 ± 123.5 min	396.2 ± 71.4 min	
	NT-proBNP, Mean ± SD		2400.9 ± 1269.8 pg/mL	1601.4 ± 2443.0 pg/mL	
Howard (2023) [12]; Rehman (2025) [13] (TRACE)					The TRACE study was excluded because its population included patients receiving sGC stimulators, which could confound the clinical outcomes
	Treatment		Placebo + ERA ± PDE5i/sGC (n = 55)	Selexipag + ERA ± PDE5i/sGC (n = 53)	
	Age, Mean ± SD		49.8 ± 13.6 years	49.0 ± 14.8 years	
	Female		76.4%	66%	
	WHO Functional Class		II 74.5%	II 62.3%	
			III 25.5%	III 37.7%	
	Asian		N/A	N/A	
	PAH Etiology		Idiopathic/Heritable 76.4%	Idiopathic/Heritable 75.5%	
			Connective tissue disease 18.2%	Connective tissue disease 15.1%	
			Congenital heart disease 1.8%	Congenital heart disease 7.5%	
			Drug-/toxin 1.8%	Drug-/toxin 1.9%	
			HIV 1.8%	HIV 1.9%	
	Background Therapy		ERA 1.8%	ERA 1.9%	
			ERA+PDE5i/sGC 98.2%	ERA+PDE5i/sGC 98.1%	
	6-MWD, Mean ± SD		449.5 ± 98.9 min	453.1 ± 129.7 min	
	NT-proBNP, Median (IQR)		207.0 (36–9811) pg/mL	16.02 (16–3871) pg/mL	

PAH, pulmonary arterial hypertension; WHO, World Health Organization; ERA, endothelin receptor antagonist; PDE5i, phosphodiesterase-5 inhibitor; sGC, soluble guanylate cyclase stimulator; 6-MWD, six-minute walk distance; NT-proBNP, N-terminal pro–B-type natriuretic peptide; mean ± SD, mean ± standard deviation; median (IQR), median (interquartile range); min–max, minimum–maximum; N/A, not available.

### 3.2 Meta-analysis and Indirect Comparison

#### 3.2.1 Primary Outcome (Composite Endpoint of Clinical Worsening)

**Fig. 2.**
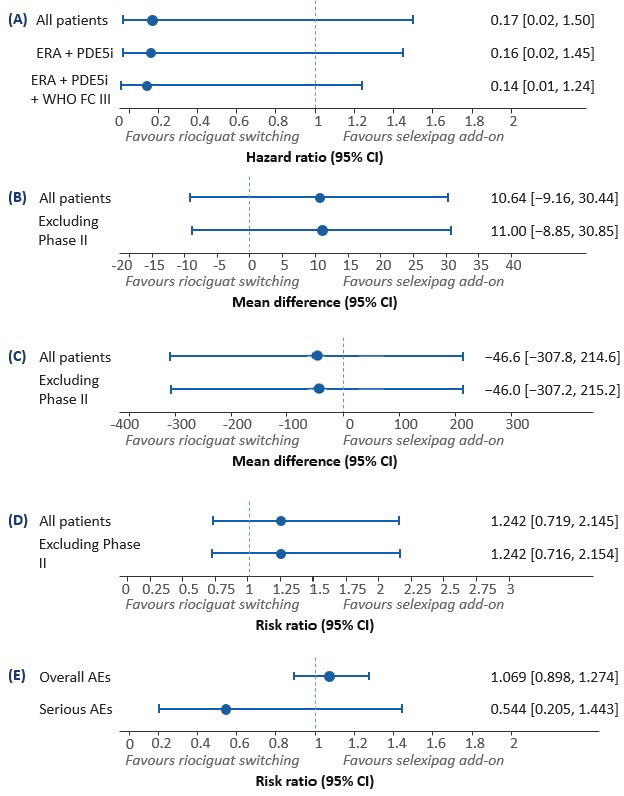
**Forest plots for clinical efficacy and safety outcomes**. Forest plot for composite endpoint of clinical worsening (A), 6-minute walking distance at 6 months (B), change in NT-proBNP at 6 months (C), improvement in WHO functional class (D), and overall and severe adverse event rate (E).

For the composite endpoint of clinical worsening, riociguat demonstrated an HR of 0.10 (95% confidence interval [CI]: 0.01–0.79) in the REPLACE study, whereas selexipag showed an HR of 0.60 (95% CI: 0.49–0.73) in the GRIPHON study. An indirect comparison yielded an HR of 0.167 (95% CI: 0.019–1.495, *p* = 0.107), which was not statistically significant. A sensitivity analysis was conducted using a subset of GRIPHON patients selected to match the baseline characteristics of the REPLACE study, including patients receiving baseline ERA+PDE5i therapy (HR: 0.159, 95% CI: 0.017–1.452, *p* = 0.107) and those classified as WHO FC III (HR: 0.135, 95% CI: 0.015–1.244, *p* = 0.074), also showed no statistically significant differences (Table 3, Fig. 2).

**Table 3.** **Results of indirect treatment comparison analysis for the composite endpoint related to clinical worsening**.

Outcomes		Treatment	Clinical trials	Riociguat replacement	Selexipag add-on	Indirect comparison of relative treatment effects (95% CI)
				Treatment effect estimate (95% CI)	Treatment effect estimate (95% CI)	
Composite endpoint of clinical worsening	All Patients	Riociguat replacement	REPLACE study	0.10 (0.01, 0.79)		0.167
						(0.019, 1.495)
		Selexipag add-on	GRIPHON study		0.60 (0.49, 0.73)	*p* = 0.1066
	ERA + PDE5i combination	Riociguat replacement	REPLACE study	0.10 (0.01, 0.79)		0.159
						(0.017, 1.452)
		Selexipag add-on	GRIPHON study		0.63 (0.44, 0.90)	*p* = 0.1069
	ERA + PDE5i combination + WHO functional class III	Riociguat replacement	REPLACE study	0.10 (0.01, 0.79)		0.135
						(0.015, 1.244)
		Selexipag add-on	GRIPHON study		0.74 (0.50, 1.10)	*p* = 0.0741

#### 3.2.2 Secondary Outcomes

6MWD: At 6 months, riociguat improved 6MWD by 23.00 m (95% CI: 5.00 to 41.00), whereas selexipag showed improvements of 12.00 m (95% CI: 3.63 to 20.37) in GRIPHON and 24.20 m (95% CI: –23.70 to 72.10) in the phase II study. The meta-analysis for selexipag yielded a mean improvement of 12.36 m (95% CI: 4.12 to 20.61). Indirect comparison showed no significant difference (MD: +10.64 m; 95% CI: –9.16 to 30.44, *p* = 0.292). Excluding the phase II study, the difference remained non-significant (MD: +11 m; 95% CI: –8.85 to 30.85, *p* = 0.270).

NT-proBNP Change: Riociguat reduced NT-proBNP by –170 pg/mL (95% CI: –426 to –86), while selexipag decreased NT-proBNP by –123 pg/mL (95% CI: –175 to –71) in GRIPHON and –212.80 pg/mL (95% CI: –1012.10 to 586.50) in the phase II study. The combined meta-analysis for selexipag was –123.38 pg/mL (95% CI: –175.27 to –71.49). Indirect comparison revealed no significant difference (MD: –46.62 pg/mL; 95% CI: –307.83 to 214.59, *p* = 0.726). Excluding the phase II study, the result remained non-significant (MD: –46 pg/mL; 95% CI: –307.23 to 215.23, *p* = 0.730).

WHO FC Improvement: Riociguat had an RR of 1.95 (95% CI: 1.27 to 3.00), and selexipag showed an RR of 1.57 (95% CI: 1.11 to 2.21) in GRIPHON and 1.52 (95% CI: 0.20 to 11.50) in the phase II study. The meta-analysis RR for selexipag was 1.57 (95% CI: 1.12 to 2.20). Indirect comparison yielded an RR of 1.24 (95% CI: 0.72 to 2.15, *p* = 0.437). Excluding the phase II study, RR remained non-significant at 1.24 (95% CI: 0.72 to 2.15, *p* = 0.441).

Safety Outcomes (Overall and Serious Adverse Events): Overall adverse events showed an RR of 1.08 (95% CI: 0.91 to 1.29) for riociguat, 1.01 (95% CI: 1.00 to 1.03) for selexipag (GRIPHON), and 0.97 (95% CI: 0.83 to 1.14) for the phase II study, yielding a combined RR of 1.01 (95% CI: 1.00 to 1.03) for selexipag. An indirect comparison showed an RR of 1.07 (95% CI: 0.90 to 1.27, *p* = 0.453), although not significant. Serious adverse events showed an RR of 0.43 (95% CI: 0.20 to 0.95) for riociguat, 0.93 (95% CI: 0.82 to 1.06) for selexipag in GRIPHON, and 0.45 (95% CI: 0.16 to 1.30) in the phase II study, resulting in a meta-analysis RR of 0.79 (95% CI: 0.44 to 1.42) for selexipag. Indirect comparison yielded an RR of 0.54 (95% CI: 0.21 to 1.44, *p* = 0.2215), which was also not statistically significant (Table 4, Fig. 2).

**Table 4.** **Results of indirect treatment comparison analysis for secondary outcomes**.

Outcomes		Treatment	Clinical trials	Riociguat replacement	Selexipag add-on	Indirect comparison of relative treatment effects (95% CI)
				Treatment effect estimate (95% CI)	Treatment effect estimate	
					(95% CI)	
6-minute walking distance at 6 months	All Patients	Riociguat	REPLACE study	23.00 (5.00, 41.00)		10.64
		Selexipag	GRIPHON study		12.00 (3.63, 20.37)	(–9.158, 30.438)
			Phase 2 study_ selexipag		24.20 (–23.70, 72.10)	*p* = 0.2920
		Meta-analysis		-	12.36 (4.12, 20.61)	
		Heterogeneity test		-	χ^2^(1) = 0.24, *p* = 0.62; I^2^ = 0%	
	Excluding Phase 2 study_slexipag	Riociguat	REPLACE study	23.00 (5.00, 41.00)		11
		Selexipag	GRIPHON study		12.00 (3.63, 20.37)	(–8.851, 30.851)
		Meta-analysis		-	-	*p* = 0.2772
		Heterogeneity test		-	-	
Change in NT-proBNP at 6 months	All Patients	Riociguat	REPLACE study	–170.00 (–426.00, 86.00)		–46.62
		Selexipag	GRIPHON study		–123.00 (–175.00, –71.00)	(–307.826, 214.586)
			Phase 2 study_ selexipag		–212.80 (–1012.10, 586.50)	*p* = 0.7263
		Meta-analysis		-	–123.38 (–175.27, –71.49)	
		Heterogeneity test		-	χ^2^(1) = 0.05, *p* = 0.83; I^2^ = 0%	
	Excluding Phase 2 study_slexipag	Riociguat	REPLACE study	–170.00 (–426.00, 86.00)		–46
		Selexipag	GRIPHON study		–123.00 (–175.00, –71.00)	(–307.228, 215.228)
		Meta-analysis		-	-	*p* = 0.7298
		Heterogeneity test		-	-	
Improvement in WHO functional class	All Patients	Riociguat	REPLACE study	1.95 (1.27, 3.00)		1.242
		Selexipag	GRIPHON study		1.57 (1.11, 2.21)	(0.719, 2.145)
			Phase 2 study_ selexipag		1.52 (0.20, 11.50)	*p* = 0.4371
		Meta-analysis		-	1.57 (1.12, 2.20)	
		Heterogeneity test		-	χ^2^(1) = 0.00, *p* = 0.97; I^2^ = 0%	
	Excluding Phase 2 study_slexipag	Riociguat	REPLACE study	23.00 (5.00, 40.00)		1.242
		Selexipag	GRIPHON study		1.57 (1.11, 2.21)	(0.716, 2.154)
		Meta-analysis		-	-	*p* = 0.4406
		Heterogeneity test		-	-	
Overall adverse event rate	All Patients	Riociguat	REPLACE study	1.08 (0.91, 1.29)		1.069
		Selexipag	GRIPHON study		1.01 (1.00, 1.03)	(0.898, 1.274)
			Phase 2 study_ selexipag		0.97 (0.83, 1.14)	*p* = 0.4531
		Meta-analysis		-	1.01 (1.00, 1.03)	
		Heterogeneity test		-	χ^2^(1) = 0.29, *p* = 0.59; I^2^ = 0%	
Severe adverse event rate	All Patients	Riociguat	REPLACE study	0.43 (0.20, 0.95)		0.544
		Selexipag	GRIPHON study		0.93 (0.82, 1.06)	(0.205, 1.443)
			Phase 2 study_ selexipag		0.45 (0.16, 1.30)	*p* = 0.2215
		Meta-analysis		-	0.79 (0.44, 1.42)	
		Heterogeneity test		-	χ^2^(1) = 1.76, *p* = 0.18; I^2^ = 43%	

**Heterogeneity test:** χ^2^ denotes Cochran’s Q test for heterogeneity (df = *k*–1), and I^2^ represents the percentage of total variability across studies attributable to between-study heterogeneity rather than chance.

## 4. Discussion

PAH is a rare disease with relatively low prevalence in South Korea. However, recent demographic changes and increased diversity in underlying conditions have contributed to a rising number of diagnoses. Between 2002 and 2018, PAH prevalence increased approximately 75-fold, and incidence increased 12-fold, highlighting improved diagnostic capabilities and a growing disease burden. Associated healthcare costs increase with the number of treated patients, underscoring the need for evidence-based, cost-effective therapeutic decisions [19].

In this study, we conducted an indirect comparison to evaluate the treatment effects of riociguat replacement and selexipag add-on therapy in patients with PAH receiving PDE5i + ERA combination therapy. Our indirect comparison relied on a single common comparator (ERA + PDE5i ± placebo) linking two treatment pairs (riociguat ± ERA vs. ERA ± PDE5i vs. selexipag ± ERA ± PDE5i). Although a network meta-analysis (NMA) could technically be applied, the Bucher’s method was considered more appropriate in this context because it is simpler, more transparent, and provides stable estimates for a small network with only a single common comparator [20]. We also evaluated the assumptions of similarity and exchangeability by comparing baseline characteristics across REPLACE and GRIPHON, including age, sex, PAH etiology, WHO functional class, 6MWD, NT-proBNP, background ERA + PDE5i use, and follow-up periods (24 weeks vs. 36 months, using 24–26-week data for GRIPHON where possible).

Although REPLACE enrolled only WHO FC III patients while GRIPHON included WHO FC II–III, the ranges of 6MWD and NT-proBNP were broadly comparable. The Coghlan post-hoc analysis, reporting outcomes in patients receiving ERA + PDE5i at baseline (100%), allowed an anchored comparison more closely aligned with REPLACE.

The phase II selexipag trial (Simonneau 2012) differed in having a small sample size, markedly higher NT-proBNP levels (likely influenced by outliers) [10], and a 17-week assessment. It was included in the primary analysis to preserve information but excluded in pre-specified sensitivity analyses.

The primary analysis showed that riociguat replacement and selexipag add-on therapies did not differ in terms of all assessed outcomes, including the composite endpoint of clinical worsening, 6MWD, NT-proBNP levels, WHO FC improvement rates, and overall and serious adverse event rates.

Despite the lack of statistical significance, riociguat replacement therapy showed a numerical trend toward greater efficacy in preventing clinical worsening than selexipag add-on therapy. However, the CIs were wide, reflecting the limited number of included studies, low event rates, and relatively small sample sizes. The HR for clinical worsening (0.167; 95% CI: 0.019–1.495) did not meet the non-inferiority margin of 1.185, which was calculated using the FDA fixed-margin method [21], suggesting that firm conclusions regarding non-inferiority cannot be drawn.

Our findings are consistent with those of Ornstová *et al*. [22], who reported no significant differences between riociguat and selexipag in an indirect comparison. However, their analysis was limited to WHO FC improvement and 6MWD as outcome measures, whereas we employed a composite endpoint of clinical worsening as the primary outcome. Traditionally, PAH clinical trials have relied on single clinical measures, such as 6MWD, as primary endpoints. Recently, however, there has been a shift toward composite endpoints reflecting clinical worsening or improvement [23]. Accordingly, we adopted a composite endpoint in our ITC to align with current trends and capture the overall clinical impact.

Our study included a more comprehensive set of RCTs and conducted sensitivity analyses focusing on patients receiving background ERA + PDE5i therapy and those classified as WHO FC III. These analyses are aligned with current PAH treatment guidelines, which suggest that patients receiving ERA + PDE5i therapy who experience clinical worsening may be considered for riociguat switching or selexipag add-on therapy [1]. We acknowledge that neither therapy demonstrated statistical superiority. However, the numerical trend toward a greater benefit with riociguat switching provides supportive information for clinical decision-making. In practice, this trend, combined with the mechanistic rationale (riociguat as a PDE5i replacement vs. selexipag as an add-on to augment the prostacyclin pathway) and patient-specific factors such as tolerability, pill burden, comorbidities, and cost, may help guide individualized therapeutic sequencing, particularly in patients who remain inadequately controlled on background ERA + PDE5i therapy.

Indirect comparisons have evolved from conservative approaches, which recommend their use only when direct RCTs are absent or compromised in internal/external validity [24, 25], to more flexible approaches that allow indirect comparisons when direct evidence is unavailable, as advocated by the National Institute for Health and Care Excellence (England) in 2013 [26] and the Canadian Agency for Drugs and Technologies in Health (Canada) in 2006 [27]. In 2016, the Pharmaceutical Benefits Advisory Committee (Australia) [28] emphasized rigorous verification of the exchangeability assumption and detailed reporting procedures. In Korea, the 2014 Health Insurance Review and Assessment Service guidelines similarly recommend indirect comparisons when direct RCT evidence is unavailable. This study met the fundamental prerequisites for indirect comparison—a systematic literature review and meta-analysis—to ensure similarity and consistency among the included studies [29]. Nevertheless, these results should be interpreted with caution due to the inability to directly validate consistency between indirect and direct comparisons.

## 5. Limitations

This study has some limitations. REPLACE was an open-label study, which may have influenced symptom reporting or motivation; however, independent assessors and multiple objective endpoints were used to mitigate this risk. Furthermore, the RCTs included in this study were relatively few, resulting in wide CIs due to low event rates and varying follow-up durations. Differences in patient severity levels also present a limitation. Further direct comparative RCTs and long-term follow-up studies are needed to clarify the relative efficacy and safety of the two therapies, providing more robust evidence to inform optimal treatment strategies for patients with PAH.

Additional comparisons restricted to patients on baseline ERA + PDE5i and to those in WHO FC III likewise showed similar HR directions but wide confidence intervals that crossed unity, underscoring that no firm conclusion on superiority or inferiority can be drawn. Moreover, GRIPHON estimated HRs for clinical worsening using a proportional hazards model over 36 months, and our indirect comparison implicitly assumes that this HR is approximately constant over time when contrasted with the 24-week HR from REPLACE; if hazards were in fact time-dependent (e.g., early event clustering or delayed treatment effects), time-scale differences could bias the comparison. Finally, the absence of individual patient-level data prevented adjustment for important prognostic factors, dosing and adherence to background therapy, and potential confounding by indication. For example, patients switched to riociguat may have had more severe disease at baseline than those who remained on PDE5i and received selexipag add-on. Therefore, these limitations reinforce the need for adequately powered head-to-head RCTs.

## 6. Conclusion

In conclusion, this indirect comparison suggests that riociguat replacement and selexipag add-on therapy provide comparable clinical benefits for patients with PAH who remain inadequately managed with PDE5i+ERA therapy. Given the current limited evidence, therapeutic decisions should be individualized, considering patient characteristics, risk profiles, drug mechanisms, and reimbursement policies. Continued research is essential to build a more robust evidence base and optimize PAH management strategies.

## Data Availability

All data used in this systematic review were extracted from publicly available, peer-reviewed articles. No new data were generated for this study. All included publications are cited in the manuscript, and the data are available within the cited articles.

## Abbreviations

ERA, Endothelin receptor antagonist; HR, Hazard ratio; ITC, Indirect treatment comparison; MD, Mean differences; PAH, Pulmonary arterial hypertension; PH, Pulmonary hypertension; PICO, Participants/Intervention/Comparator/Outcome; RCT, Randomized controlled trial; RR, Risk ratio.
