# Supplementary material for: Indirect Treatment Comparison of Riociguat Replacement Therapy and Selexipag Add-on Therapy in Patients With Pulmonary Arterial Hypertension: Results From a Systematic Review
Source: Rev Cardiovasc Med. 2026 May 27;27(5):46524. doi: 10.31083/RCM46524 (PMC13227382; doi:10.31083/RCM46524)
Supplement: Supplementary file 1 [file 2153-8174-27-5-46524-s1.zip › Supplementary Material.docx]

Supplementary Table 1-a. PRISMA 2020 for Abstracts Checklist

| **Section and Topic** | **Item #** | **Checklist item** | **Reported (Yes/No)** |
| --- | --- | --- | --- |
| **TITLE** | | |  |
| Title | 1 | Identify the report as a systematic review. | Yes |
| **BACKGROUND** | | |  |
| Objectives | 2 | Provide an explicit statement of the main objective(s) or question(s) the review addresses. | Yes |
| **METHODS** | | |  |
| Eligibility criteria | 3 | Specify the inclusion and exclusion criteria for the review. | Yes |
| Information sources | 4 | Specify the information sources (e.g. databases, registers) used to identify studies and the date when each was last searched. | Yes |
| Risk of bias | 5 | Specify the methods used to assess risk of bias in the included studies. | Yes |
| Synthesis of results | 6 | Specify the methods used to present and synthesise results. | Yes |
| **RESULTS** | | |  |
| Included studies | 7 | Give the total number of included studies and participants and summarise relevant characteristics of studies. | Yes |
| Synthesis of results | 8 | Present results for main outcomes, preferably indicating the number of included studies and participants for each. If meta-analysis was done, report the summary estimate and confidence/credible interval. If comparing groups, indicate the direction of the effect (i.e. which group is favoured). | Yes |
| **DISCUSSION** | | |  |
| Limitations of evidence | 9 | Provide a brief summary of the limitations of the evidence included in the review (e.g. study risk of bias, inconsistency and imprecision). | Yes |
| Interpretation | 10 | Provide a general interpretation of the results and important implications. | Yes |
| **OTHER** | | |  |
| Funding | 11 | Specify the primary source of funding for the review. | Yes |
| Registration | 12 | Provide the register name and registration number. | No |

*From:*  Page MJ, McKenzie JE, Bossuyt PM, Boutron I, Hoffmann TC, Mulrow CD, et al. The PRISMA 2020 statement: an updated guideline for reporting systematic reviews. BMJ 2021;372:n71. doi: 10.1136/bmj.n71

Supplementary Table 1-b. PRISMA 2020 Checklist

| **Section and Topic** | **Item #** | **Checklist item** | **Location where item is reported** |
| --- | --- | --- | --- |
| **TITLE** | | |  |
| Title | 1 | Identify the report as a systematic review. | Page 1 |
| **ABSTRACT** | | |  |
| Abstract | 2 | See the PRISMA 2020 for Abstracts checklist. | Page 1 |
| **INTRODUCTION** | | |  |
| Rationale | 3 | Describe the rationale for the review in the context of existing knowledge. | Page 2 |
| Objectives | 4 | Provide an explicit statement of the objective(s) or question(s) the review addresses. | Page 2 |
| **METHODS** | | |  |
| Eligibility criteria | 5 | Specify the inclusion and exclusion criteria for the review and how studies were grouped for the syntheses. | Page 3 |
| Information sources | 6 | Specify all databases, registers, websites, organisations, reference lists and other sources searched or consulted to identify studies. Specify the date when each source was last searched or consulted. | Page 2-3 |
| Search strategy | 7 | Present the full search strategies for all databases, registers and websites, including any filters and limits used. | Supplement |
| Selection process | 8 | Specify the methods used to decide whether a study met the inclusion criteria of the review, including how many reviewers screened each record and each report retrieved, whether they worked independently, and if applicable, details of automation tools used in the process. | Page 3 |
| Data collection process | 9 | Specify the methods used to collect data from reports, including how many reviewers collected data from each report, whether they worked independently, any processes for obtaining or confirming data from study investigators, and if applicable, details of automation tools used in the process. | Page 3 |
| Data items | 10a | List and define all outcomes for which data were sought. Specify whether all results that were compatible with each outcome domain in each study were sought (e.g. for all measures, time points, analyses), and if not, the methods used to decide which results to collect. | Page 3 |
|  | 10b | List and define all other variables for which data were sought (e.g. participant and intervention characteristics, funding sources). Describe any assumptions made about any missing or unclear information. | Page 3 |
| Study risk of bias assessment | 11 | Specify the methods used to assess risk of bias in the included studies, including details of the tool(s) used, how many reviewers assessed each study and whether they worked independently, and if applicable, details of automation tools used in the process. | Page 3 |
| Effect measures | 12 | Specify for each outcome the effect measure(s) (e.g. risk ratio, mean difference) used in the synthesis or presentation of results. | Page 3-4 |
| Synthesis methods | 13a | Describe the processes used to decide which studies were eligible for each synthesis (e.g. tabulating the study intervention characteristics and comparing against the planned groups for each synthesis (item #5)). | Page 3-4 |
|  | 13b | Describe any methods required to prepare the data for presentation or synthesis, such as handling of missing summary statistics, or data conversions. | Page 3-4 |
|  | 13c | Describe any methods used to tabulate or visually display results of individual studies and syntheses. | Page 3-4 |
|  | 13d | Describe any methods used to synthesize results and provide a rationale for the choice(s). If meta-analysis was performed, describe the model(s), method(s) to identify the presence and extent of statistical heterogeneity, and software package(s) used. | Page 3-4 |
|  | 13e | Describe any methods used to explore possible causes of heterogeneity among study results (e.g. subgroup analysis, meta-regression). | Page 3-4 |
|  | 13f | Describe any sensitivity analyses conducted to assess robustness of the synthesized results. | Page 3-4 |
| Reporting bias assessment | 14 | Describe any methods used to assess risk of bias due to missing results in a synthesis (arising from reporting biases). | Page 3 |
| Certainty assessment | 15 | Describe any methods used to assess certainty (or confidence) in the body of evidence for an outcome. | Certainty assessment was not performed. |
| **RESULTS** | | |  |
| Study selection | 16a | Describe the results of the search and selection process, from the number of records identified in the search to the number of studies included in the review, ideally using a flow diagram. | Page 4 |
|  | 16b | Cite studies that might appear to meet the inclusion criteria, but which were excluded, and explain why they were excluded. | Page 4 |
| Study characteristics | 17 | Cite each included study and present its characteristics. | Page 4-9 |
| Risk of bias in studies | 18 | Present assessments of risk of bias for each included study. | Page 9 |
| Results of individual studies | 19 | For all outcomes, present, for each study: (a) summary statistics for each group (where appropriate) and (b) an effect estimate and its precision (e.g. confidence/credible interval), ideally using structured tables or plots. | Page 9-10 |
| Results of syntheses | 20a | For each synthesis, briefly summarise the characteristics and risk of bias among contributing studies. | Page 5-9 |
|  | 20b | Present results of all statistical syntheses conducted. If meta-analysis was done, present for each the summary estimate and its precision (e.g. confidence/credible interval) and measures of statistical heterogeneity. If comparing groups, describe the direction of the effect. | Page 7-8 |
|  | 20c | Present results of all investigations of possible causes of heterogeneity among study results. | Not reported |
|  | 20d | Present results of all sensitivity analyses conducted to assess the robustness of the synthesized results. | Sensitivity analysis was not performed. |
| Reporting biases | 21 | Present assessments of risk of bias due to missing results (arising from reporting biases) for each synthesis assessed. | Not reported |
| Certainty of evidence | 22 | Present assessments of certainty (or confidence) in the body of evidence for each outcome assessed. | Certainty assessment was not performed. |
| **DISCUSSION** | | |  |
| Discussion | 23a | Provide a general interpretation of the results in the context of other evidence. | Page 10-11 |
|  | 23b | Discuss any limitations of the evidence included in the review. | Page 11-12 |
|  | 23c | Discuss any limitations of the review processes used. | Not reported |
|  | 23d | Discuss implications of the results for practice, policy, and future research. | Page 12 |
| **OTHER INFORMATION** | | |  |
| Registration and protocol | 24a | Provide registration information for the review, including register name and registration number, or state that the review was not registered. | Page 2 |
|  | 24b | Indicate where the review protocol can be accessed, or state that a protocol was not prepared. | Page 2 |
|  | 24c | Describe and explain any amendments to information provided at registration or in the protocol. | No amendments |
| Support | 25 | Describe sources of financial or non-financial support for the review, and the role of the funders or sponsors in the review. | Page 12 |
| Competing interests | 26 | Declare any competing interests of review authors. | Page 12 |
| Availability of data, code and other materials | 27 | Report which of the following are publicly available and where they can be found: template data collection forms; data extracted from included studies; data used for all analyses; analytic code; any other materials used in the review. | Page 12 |

*From:*  Page MJ, McKenzie JE, Bossuyt PM, Boutron I, Hoffmann TC, Mulrow CD, et al. The PRISMA 2020 statement: an updated guideline for reporting systematic reviews. BMJ 2021;372:n71. doi: 10.1136/bmj.n71

For more information, visit: <http://www.prisma-statement.org/>

Supplementary Table 2-a. Literature Search Strategy of PubMed for Riociguat

|  | Query |
| --- | --- |
| 1 | "hypertension, pulmonary"[MeSH Terms] |
| 2 | "pulmonary hypertension"[Title/Abstract] |
| 3 | "pulmonary arterial hypertension"[Title/Abstract] |
| 4 | PAH[Title/Abstract] |
| 5 | #1 OR #2 OR #3 OR #4 |
| 6 | (((("riociguat" [Supplementary Concept]) OR (riociguat)) OR (Adempas)) OR ("BAY 63-2521")) OR ("625115-55-1") |
| 7 | (((((((randomized controlled trial[Publication Type]) OR (controlled clinical trial[Publication Type])) OR ((randomized[Title/Abstract]) AND (placebo[Title/Abstract]))) OR ((drug therapy[MeSH Subheading]))) OR (randomly[Title/Abstract])) OR (trial[Title/Abstract])) OR (groups[Title/Abstract])) NOT (((animals[MeSH Terms])) NOT (humans[MeSH Terms])) |
| 8 | #5 AND #6 AND #7 |

Supplementary Table 2-b. Literature Search Strategy of Cochrane Library for Riociguat

|  | Query |
| --- | --- |
| 1 | MeSH descriptor: [Hypertension, Pulmonary] explode all trees |
| 2 | (pulmonary NEAR/3 hypertension):ti,ab,kw |
| 3 | (PAH):ti,ab,kw |
| 4 | #1 OR #2 OR #3 |
| 5 | (riociguat):ti,ab,kw |
| 6 | (Adempas):ti,ab,kw |
| 7 | ("BAY 63-2521"):ti,ab,kw |
| 8 | (625115551):ti,ab,kw |
| 9 | #5 OR #6 OR #7 OR #8 |
| 10 | #4 AND #9 |
| 11 | MeSH descriptor: [Random Allocation] explode all trees |
| 12 | MeSH descriptor: [Randomized Controlled Trials as Topic] explode all trees |
| 13 | MeSH descriptor: [Double-Blind Method] explode all trees |
| 14 | MeSH descriptor: [Single-Blind Method] explode all trees |
| 15 | MeSH descriptor: [Clinical Trials as Topic] explode all trees |
| 16 | MeSH descriptor: [Placebos] explode all trees |
| 17 | ("randomly allocated"):ti,ab,kw |
| 18 | (placebo*):ti,ab,kw |
| 19 | (allocated NEAR/2 random*):ti,ab,kw |
| 20 | (randomi?ed controlled trial*):ti,ab,kw |
| 21 | #11 OR #12 OR #13 OR #14 OR #15 OR #16 OR #17 OR #18 OR #19 OR #20 |
| 22 | #10 AND #21 |

Supplementary Table 2-c. Literature Search Strategy of EMBASE for Riociguat

|  | Query |
| --- | --- |
| 1 | exp pulmonary hypertension/ |
| 2 | (pulmonary adj3 hypertension).mp. [mp=title, abstract, heading word, drug trade name, original title, device manufacturer, drug manufacturer, device trade name, keyword heading word, floating subheading word, candidate term word] |
| 3 | PAH.mp. |
| 4 | 1 or 2 or 3 |
| 5 | exp riociguat/ |
| 6 | riociguat.mp. |
| 7 | Adempas.mp. |
| 8 | BAY 632521.mp. |
| 9 | BAY 63-2521.mp. |
| 10 | 5 or 6 or 7 or 8 or 9 |
| 11 | (Randomized Controlled Trials as Topic/ or randomized controlled trial/ or Random Allocation/ or Double Blind Method/ or Single Blind Method/ or clinical trial/ or clinical trial, phase i.pt. or clinical trial, phase ii.pt. or clinical trial, phase iii.pt. or clinical trial, phase iv.pt. or controlled clinical trial.pt. or randomized controlled trial.pt. or multicenter study.pt. or clinical trial.pt. or exp Clinical Trials as topic/ or (clinical adj trial$).tw. or ((singl$ or doubl$ or treb$ or tripl$) adj (blind$3 or mask$3)).tw. or PLACEBOS/ or placebo$.tw. or randomly allocated.tw. or (allocated adj2 random$).tw.) not (case report.tw. or letter/ or historical article/) |
| 12 | 4 and 10 and 11 |

Supplementary Table 2-d. Literature Search Strategy of PubMed for selexipag

|  | Query |
| --- | --- |
| 1 | "hypertension, pulmonary"[MeSH Terms] |
| 2 | "pulmonary hypertension"[Title/Abstract] |
| 3 | "pulmonary arterial hypertension"[Title/Abstract] |
| 4 | PAH[Title/Abstract] |
| 5 | #1 OR #2 OR #3 OR #4 |
| 6 | ("selexipag" [Supplementary Concept]) OR ((((((uptravi) OR (NS-304)) OR (NS304)) OR (ACT293987)) OR (ACT-293987)) OR (5EXC0E384L)) |
| 7 | (((((((randomized controlled trial[Publication Type]) OR (controlled clinical trial[Publication Type])) OR ((randomized[Title/Abstract]) AND (placebo[Title/Abstract]))) OR ((drug therapy[MeSH Subheading]))) OR (randomly[Title/Abstract])) OR (trial[Title/Abstract])) OR (groups[Title/Abstract])) NOT (((animals[MeSH Terms])) NOT (humans[MeSH Terms])) |
| 8 | #5 AND #6 AND #7 |

Supplementary Table 2-e. Literature Search Strategy of Cochrane Library for Selexipag

|  | Query |
| --- | --- |
| 1 | MeSH descriptor: [Hypertension, Pulmonary] explode all trees |
| 2 | (pulmonary NEAR/3 hypertension):ti,ab,kw |
| 3 | (PAH):ti,ab,kw |
| 4 | #1 OR #2 OR #3 |
| 5 | (selexipag):ti,ab,kw |
| 6 | (uptravi):ti,ab,kw |
| 7 | ("NS 304"):ti,ab,kw |
| 8 | ("ACT 293987"):ti,ab,kw |
| 9 | #5 OR #6 OR #7 OR #8 |
| 10 | #4 AND #9 |
| 11 | MeSH descriptor: [Random Allocation] explode all trees |
| 12 | MeSH descriptor: [Randomized Controlled Trials as Topic] explode all trees |
| 13 | MeSH descriptor: [Double-Blind Method] explode all trees |
| 14 | MeSH descriptor: [Single-Blind Method] explode all trees |
| 15 | MeSH descriptor: [Clinical Trials as Topic] explode all trees |
| 16 | MeSH descriptor: [Placebos] explode all trees |
| 17 | ("randomly allocated"):ti,ab,kw |
| 18 | (placebo*):ti,ab,kw |
| 19 | (allocated NEAR/2 random*):ti,ab,kw |
| 20 | (randomi?ed controlled trial*):ti,ab,kw |
| 21 | #11 OR #12 OR #13 OR #14 OR #15 OR #16 OR #17 OR #18 OR #19 OR #20 |
| 22 | #10 AND #21 |

Supplementary Table 2-f. Literature Search Strategy of EMBASE for Selexipag

|  | Query |
| --- | --- |
| 1 | exp pulmonary hypertension/ |
| 2 | (pulmonary adj3 hypertension).mp. [mp=title, abstract, heading word, drug trade name, original title, device manufacturer, drug manufacturer, device trade name, keyword heading word, floating subheading word, candidate term word] |
| 3 | PAH.mp. |
| 4 | 1 or 2 or 3 |
| 5 | exp selexipag/ |
| 6 | selexipag.mp. |
| 7 | uptravi.mp. |
| 8 | NS-304.mp. |
| 9 | "ACT 293987".mp. |
| 10 | 5 or 6 or 7 or 8 or 9 |
| 11 | (Randomized Controlled Trials as Topic/ or randomized controlled trial/ or Random Allocation/ or Double Blind Method/ or Single Blind Method/ or clinical trial/ or clinical trial, phase i.pt. or clinical trial, phase ii.pt. or clinical trial, phase iii.pt. or clinical trial, phase iv.pt. or controlled clinical trial.pt. or randomized controlled trial.pt. or multicenter study.pt. or clinical trial.pt. or exp Clinical Trials as topic/ or (clinical adj trial$).tw. or ((singl$ or doubl$ or treb$ or tripl$) adj (blind$3 or mask$3)).tw. or PLACEBOS/ or placebo$.tw. or randomly allocated.tw. or (allocated adj2 random$).tw.) not (case report.tw. or letter/ or historical article/) |
| 12 | 4 and 10 and 11 |

Supplementary Table 3. Summary of the Risk of Bias Assessment Results for Candidate Clinical Trials for Indirect Comparison

| First author (year)  Trial name | Random Sequence Generation | Allocation Concealment | Blinding of Participants and Personnel | Blinding of Outcome Assessment | Incomplete Outcome Data | Selective Reporting | Other Bias |
| --- | --- | --- | --- | --- | --- | --- | --- |
| Hooper (2021)  REPLACE study | Low | Low | High  (open label) | Low | Low | Low | Low |
| Sitbon (2015)  GRIPHON study | Low | Low | Low | Low | Low | Low | Low |
| Coghlan (2018)  GRIPHON study | Low | Low | Low | Low | Low | Low | Low |
| Simonneau (2012)  Phase II study | Low | Low | Low | Unclear Risk (protocol not found) | Low | Low | High  (single-center study) |


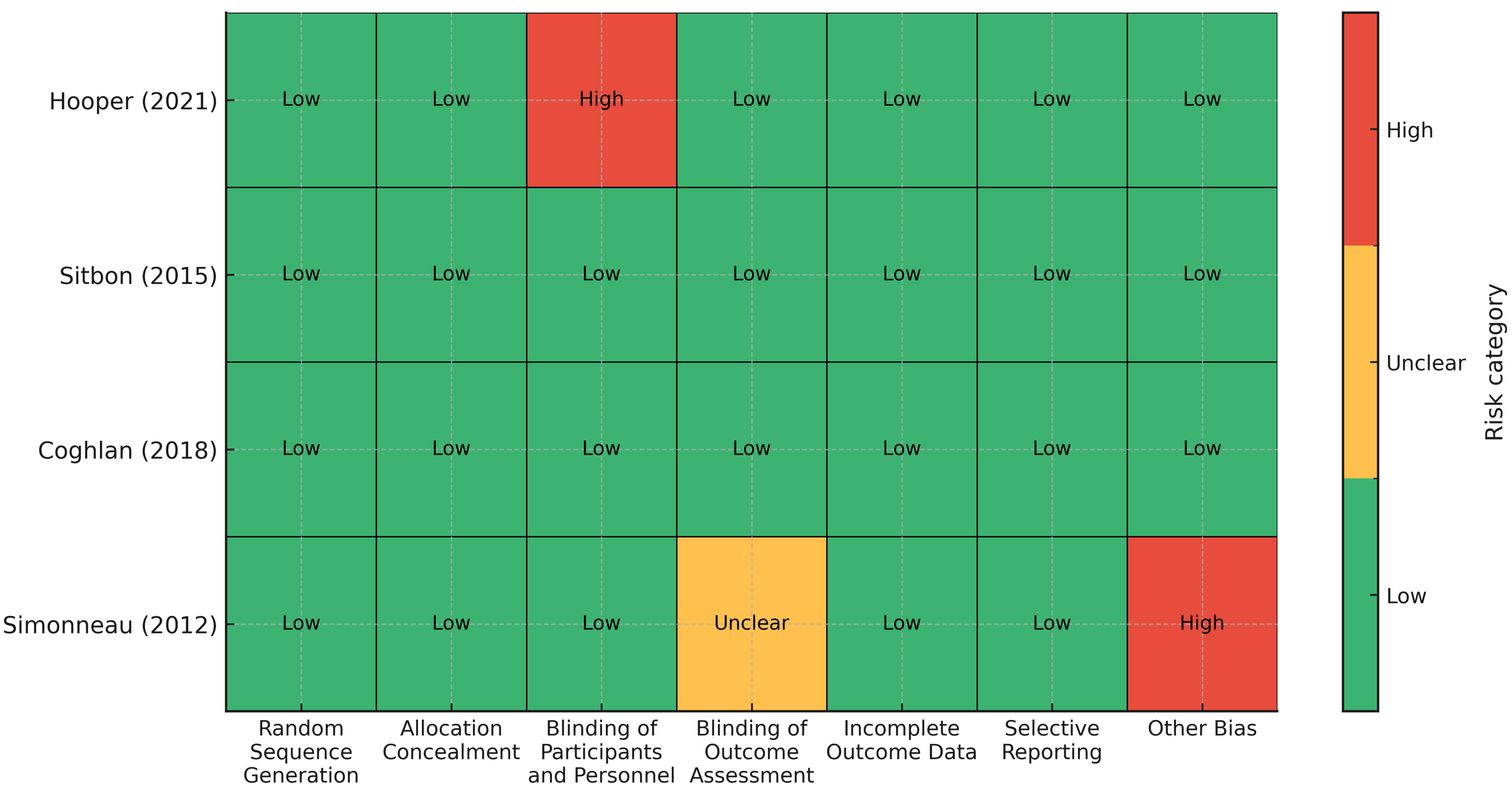


Supplementary Fig. 1. Cochrane Risk of Bias 2.0 Summary Heatmap
